# Glucose induced activation of canonical Wnt signaling pathway in hepatocellular carcinoma is regulated by DKK4

**DOI:** 10.1038/srep27558

**Published:** 2016-06-08

**Authors:** Surbhi Chouhan, Snahlata Singh, Dipti Athavale, Pranay Ramteke, Vimal Pandey, Jomon Joseph, Rajashekar Mohan, Praveen Kumar Shetty, Manoj Kumar Bhat

**Affiliations:** 1 National Centre for Cell Science, Savitribai Phule Pune University Campus, Ganeshkhind, Pune-411 007, India; 2 Laboratory of Neuroscience, Department of Biotechnology and Bioinformatics, Hyderabad Central University, Hyderabad-500 046, India; 3 Sri Dharmasthala Manjunatheshwara Medical Sciences and Hospital, Dharwad-580009, Karnataka, India

## Abstract

Elevated glycemic index, an important feature of diabetes is implicated in an increased risk of hepatocellular carcinoma (HCC). However, the underlying molecular mechanisms of this association are relatively less explored. Present study investigates the effect of hyperglycemia over HCC proliferation. We observed that high glucose culture condition (HG) specifically activates canonical Wnt signaling in HCC cells, which is mediated by suppression of DKK4 (a Wnt antagonist) expression and enhanced β-catenin level. Functional assays demonstrated that a normoglycemic culture condition (NG) maintains constitutive expression of DKK4, which controls HCC proliferation rate by suppressing canonical Wnt signaling pathway. HG diminishes DKK4 expression leading to loss of check at G0/G1/S phases of the cell cycle thereby enhancing HCC proliferation, in a β-catenin dependent manner. Interestingly, in NOD/SCID mice supplemented with high glucose, HepG2 xenografted tumors grew rapidly in which elevated levels of β-catenin, c-Myc and decreased levels of DKK4 were detected. Knockdown of DKK4 by shRNA promotes proliferation of HCC cells in NG, which is suppressed by treating cells exogenously with recombinant DKK4 protein. Our *in vitro* and *in vivo* results indicate an important functional role of DKK4 in glucose facilitated HCC proliferation.

Hepatocellular carcinoma (HCC) is a worldwide malignancy and the incidence rates have increased significantly over the past two decades^1^. The major risk factors for development of HCC have been attributed to hepatitis virus or alcoholic liver disease, which corresponds to 50% of total incidences^2^. Other risk factors include extensive alcohol consumption, nonalcoholic steatohepatitis, cirrhosis and exposure to aflatoxin B^3^. However, in 15–30% of HCC patients, no specific risk factor has been attributed^4^. Number of case control, cohort and retrospective observational studies indicate that diabetes mellitus (DM) is a potential risk factor for HCC and it also enhances mortality^5^,^6^,^7^. A systemic review suggests that diabetes increases the risk of HCC by 2.5 folds^8^. Diabetic liver is associated with increased cirrhosis and nonalcoholic fatty liver disease (NAFLD)^9^. NAFLD later develops into nonalcoholic steatohepatitis (NASH), which has been reported to progress into HCC^10^. The diabetes-cancer link has been hypothesized to rely on factors such as hormones (insulin, IGF-1, adipokines, etc.), immunoresponse (inflammation) or metabolic features (hyperglycemia)^11^. So far, insulin has been considered as a major link between diabetes and cancer, while high glucose has been considered as a subordinate cause^12^. However, recent epidemiological studies strongly link high glycemic index to HCC risk^13^,^14^,^15^, which suggests that glucose homeostasis directly affects cancer associated pathways.

Recent studies report that aberrant Wnt signaling pathway is present in 40–90% gastrointestinal cancers including HCC^16^,^17^,^18^,^19^. These are the specific cancer sites more tightly associated with metabolic parameters altered in diabetes. Also, mutations in the CTNNB1 gene (encodes β-catenin) and atypical accumulation of β-catenin protein has been reported in human HCC tumors^20^. Moreover, growing number of evidences suggest that canonical Wnt signaling, which is modulated by β-catenin, may serve as a pathway that links enhanced cancer risk with altered metabolic state, such as in hyperglycemia^21^,^22^,^23^,^24^,^25^,^26^,^27^. Currently, direct association between involvement of high glucose induced Wnt signaling and HCC growth, is the least explored.

Canonical Wnt signaling is suppressed by dickkopf (DKK) family of secretory glycoproteins namely DKK1, DKK2, DKK3 and DKK4^28^. DKK proteins bind to low-density lipoprotein receptor-related protein-5 (LRP 5) which enhances GSK3β mediated degradation of β-catenin complex in the cytoplasm and reducing transcription of target genes^29^. Contradictorily, a report suggests that DKK1 is associated with increased β-catenin accumulation^30^ while DKK2 and DKK3 genes are inactive in HCC tumors because of epigenetic modification^31^. Although, reduced expression of DKK4 has only been reported in HCC cell lines and human HCC tumors^32^, its functional relevance under hyperglycemia is still unexplored. Present study investigates the role of DKK4 in glucose induced proliferation of HCC cells through modulation of canonical Wnt signaling pathway.

## Results

### High glucose enhances proliferation in HCC by increasing percent of cells in S phase

**Figure 1:**
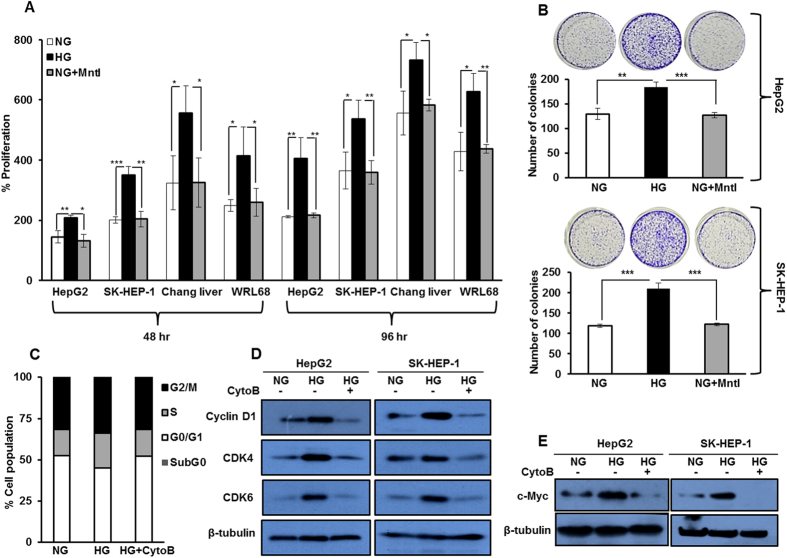
Glucose enhances proliferation in hepatocellular carcinoma cell lines. (**A**) HCC cells (HepG2, SK-HEP-1, Chang liver and WRL 68) were cultured in HG and NG conditions for 48 hr and 96 hr. Thereafter, percent proliferation was determined by MTT assay. Mannitol (Mntl) treated NG conditions served as an osmolarity control. (**B**) HCC cells were cultured in NG, NG + Mntl and HG, and colonies were visualized by crystal violet stain and counted after 21 days. (**C**) Cell cycle profile of HepG2 cells cultured in NG, HG and HG + CytoB for 16 hr. Bar graphs represent percentage of cells in different phases of cell cycle by flow cytometry of an experiment done in triplicate. (D**,E**) HepG2 cells were cultured in NG, HG and HG + CytoB for 16 hr and whole cell lysates were subjected to detection of Cyclin D1, CDK6, CDK4 and c-Myc by western blotting. All the bar graph represents the mean ± SD of an experiment done in triplicate (*P < 0.05, **P < 0.001, ***P < 0.0001). Cropped blots are used in the main figure and full length blots are included in Supplementary Figure 6.

We first investigated whether glucose directly affects HCC growth by determining percent change in proliferation of HepG2, SK-HEP-1, Chang Liver and WRL 68 cells under varying glucose culture conditions for 48 hr and 96 hr. We observed that treatment with high glucose significantly increases proliferation of HCC cells (Fig. 1A). To rule out the possibility that this effect is due to differences in the osmolarity, cells were cultured in NG along with mannitol (Mntl) (19.5 mM), as an osmolarity control. No significant change in proliferation of cells cultured in NG medium, with or without Mntl was detected, as assessed by MTT assay (Fig. 1A). Also, in the colony formation assay, significantly increased numbers of colonies were detected in HepG2 and SK-HEP-1 cells cultured in HG as compared to NG (Fig. 1B). These results indicate that HG enhances proliferation of HCC cells.

Rapidly proliferating cells require more glucose to meet their energy requirements. Active uptake of glucose is mainly dependent on the level and activity of glucose transporters^33^. Thus, we probed into the status of glucose transporter-1 (Glut-1) and glucose transporter-2 (Glut-2) by western blotting. Although, no change in their levels were detected (Supplementary Figure 1A), an increase in radioactive glucose uptake and utilization was noted in HepG2 cells in a time and concentration dependent manner (Supplementary Figure 1B,C). The maximum uptake of glucose occurs at 12 hr. These results indicate that cells cultured in HG uptake more glucose and rapidly utilize it, than cells in NG or HG treated with cytochalasin B (CytoB), an inhibitor of glucose transporter activity.

Enhanced cellular proliferation is always associated with changes in cell cycle phases and maximum uptake of nutrients, such as glucose, is associated with the synthesis phase (S-phase)^27^. Implication of increased glucose uptake and its utilization by cells was evaluated by performing cell cycle analysis. We observed that in HepG2 cells, the percentage of cells in S-phase of cell cycle was more in HG than in NG, which was curtailed by CytoB (Fig. 1C). Additionally, we checked the levels of regulatory proteins such as CDK6, CDK4, Cyclin D1 and c-Myc, specific to G_0_/G_1_/S phases of cell cycle, by western blot analysis. In HG cells, the levels of these proteins were elevated compared to NG cells and in HG cells treated with CytoB (Fig. 1D,E). These results indicate that, HCC cells cultured under variable glucose conditions show enhanced glucose uptake and proliferate more in HG than in NG or HG with CytoB, which parallels with increase in percentage of cells in S-phase of cell cycle. Data are presented as mean ± standard deviation (SD) of triplicates. Statistical comparison was performed by Student’s 2-tailed unpaired t-test. The values of P < 0.05 were considered statistically significant.

### High glucose suppresses expression of Wnt antagonist DKK4

Since changes in proliferation of HCC cells in response to variable glucose conditions were observed, we investigated the effect of glucose on various proliferative signaling cascades. In response to variable glucose culture conditions, the levels of proliferation associated proteins such as pMEK, MEK, pAKT, AKT, pERK, ERK and Raf-1 remain unaltered (Supplementary Figure 2A). However, c-Myc (a target of canonical Wnt signaling) levels were prominently increased in both HepG2 and SK-HEP-1 cells (Fig. 1E). The enhanced level of c-Myc because of glucose induced activation of canonical Wnt signaling has been previously reported^25^.

Abrupt oncogenic activation of canonical Wnt signaling pathway by Wnt3a ligand is one of the comprehensive events in hepatocarcinogenesis^17^. Also, it has been reported that the activity of Wnt ligands is suppressed by the presence of antagonists such as DKK secretory proteins^29^,^32^. Therefore, to check for correlation if any, between DKK4, β–catenin, Wnt3a and c-Myc expression in HCC, we searched the ONCOMINE human cancer genomics database. Data available from 210 samples from TCGA cohort suggest that the expression level of DKK4 was decreased while c-Myc and Wnt3a levels were increased in HCC in comparison to normal liver (Supplementary Figure 3A). Analysis of grade wise distribution (grade 1–4) of these samples showed reduction in DKK4 expression and increase in expression of c-Myc and Wnt3a (Supplementary Figure 3B), whereas, CTNNB1 expression remain unchanged. These observations prompted us to investigate the alterations in the levels of Wnt3a and DKK proteins in response to glucose.

**Figure 2:**
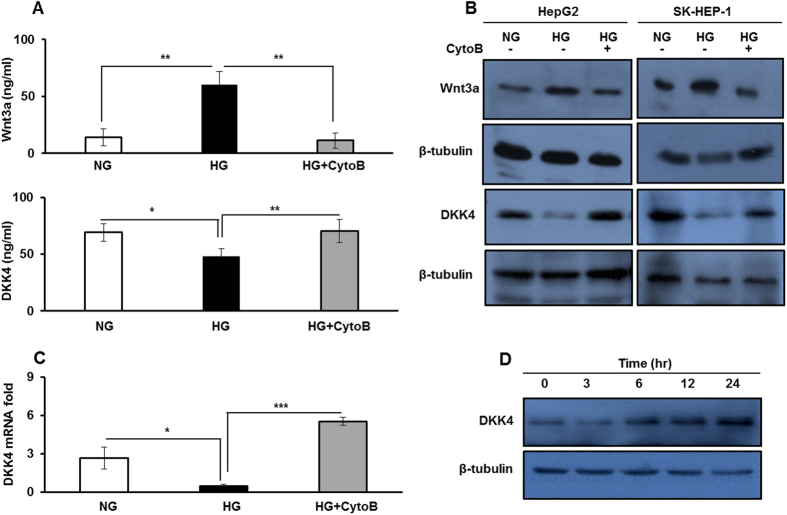
High glucose increases Wnt3a level and suppresses the expression of its antagonist DKK4. (**A**) ELISA measurements of Wnt3a and DKK4 secretory proteins in culture media collected after 16 hr from HepG2 cells in NG, HG and HG + CytoB. (**B**) HepG2 and SK-HEP-1 cells were cultured in NG, HG and HG + CytoB for 16 hr. Whole cell lysates were subjected to western blotting and levels of Wnt3a and DKK4 proteins were detected. (**C**) HepG2 cells were cultured in NG, HG and HG + CytoB for 16 hr. Total RNA was isolated and cDNA was prepared to determine relative mRNA fold expression of DKK4 by quantitative real-time RT-PCR. (**D**) HepG2 cells were cultured in HG for 16 hr and then allowed to grow in medium without glucose for indicated time course. Whole cell lysates were prepared for detection of DKK4 protein by western blotting. All the bar graphs represent the mean ± SD of an experiment done in triplicate (*P < 0.05, **P < 0.001, ***P < 0.0001). Cropped blots are used in the main figure and full length blots are included in Supplementary Figure 7.

In response to variable glucose concentrations we examined the status of Wnt3a and DKK4 ligand. Interestingly, Wnt3a level was significantly elevated in culture medium and in whole cell lysate of HepG2 cells cultured in HG compared to NG or in HG with CytoB (p < 0.005 each) (Fig. 2A,B upper panel). Also, we screened the mRNA levels of DKK genes (Supplementary Figure 2B) and observed that under HG condition, the level of only DKK4 mRNA significantly decreases than in NG (P < 0.05). DKK4 mRNA level increases again upon inhibition of glucose uptake by treating cells in HG with CytoB (P < 0.005) (Fig. 2C). Also, the level of secreted form of DKK4 decreases in HG compared to NG (P < 0.05) culture medium. Interestingly, in HG cells, upon treatment with CytoB, DKK4 secretory protein level increases than in HG alone (P < 0.005) (Fig. 2A lower panel). The level of DKK4 protein in whole cell lysate of HCC cells diminished in HG compared to NG and in HG with CytoB (Fig. 2B lower panel). On the contrary, when HepG2 cells were cultured for varying time points in glucose free medium, the level of DKK4 protein increases with increase in time duration of glucose deprivation (Fig. 2D). Additionally, supplementation of other carbon sources to cells, such as glutamine (Glt) or glucose analogue such as L-glucose (L-Glu) did not affect DKK4 protein level (Supplementary Figure 2D). These results suggest that in HG condition DKK4 expression is suppressed whereas, Wnt3a level is increased, which might be involved in activation of Wnt signaling pathway.

### High glucose induces β-catenin dependent activation of canonical Wnt signaling pathway

A reciprocal relation between DKK4 and Wnt3a ligand level is involved in β–catenin induced activation of canonical Wnt signaling pathway^32^. Therefore, we next investigated changes in β–catenin expression in HCC cells in response to glucose. The level of β-catenin transcript under NG and HG culture conditions was not altered significantly, while a decrease was observed between HG and HG with CytoB (Supplementary Figure 2C). These results suggest that glucose concentration per se has no effect on β–catenin transcript level, whereas inhibition of glucose uptake causes significant changes in its level when compared with levels in NG or HG conditions.

**Figure 3:**
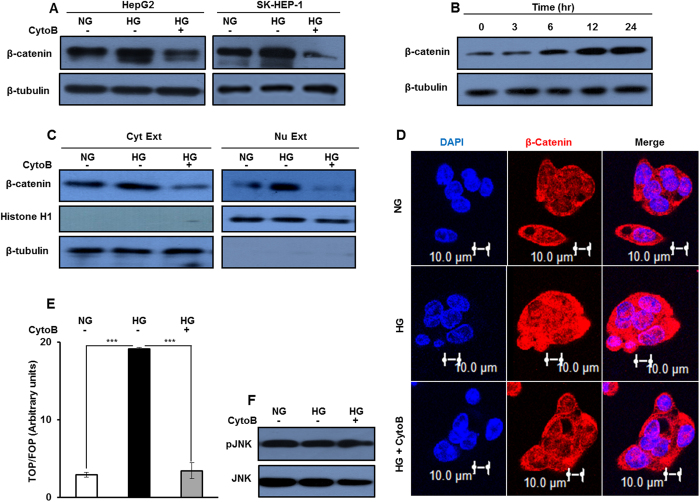
High glucose induces activation of canonical Wnt signaling. (**A–F**) HepG2 and SK-HEP-1 cells were cultured in NG, HG and HG + CytoB for 16 hr. Whole cell lysates were subjected to western blotting and levels of β–catenin, pJNK and JNK proteins were detected. (**B**) HepG2 cells were serum and glucose starved for 2 hr and then cultured in HG for indicated time intervals. Whole cell lysates were prepared and β-catenin protein level was detected by western blotting. (**C**) Cytosolic and nuclear extracts were prepared from HepG2 cells cultured in NG, HG and HG + CytoB for 16 hr and processed for immunodetection of β–catenin levels. Cropped blots are used in the main figure and full length blots are included in Supplementary Figure 8. (**D**) HepG2 cells were cultured in NG, HG and HG + CytoB for 16 hr and cells were processed for immunofluorosence based confocal imaging of β–catenin protein. Bars represent 10 μm. (**E**) β–catenin transcription activity was determined by TCF reporter assay in HepG2 cells cultured in NG, HG and HG + CytoB for 16 hr. The luciferase intensities were normalized with Renilla intensities and data is represented as ratio of TOP/FOP. Bar graph represents mean ± SE of three independent experiments (*P < 0.05, **P < 0.001, ***P < 0.0001).

Also, β-catenin protein level was increased under HG condition, which upon inhibition of glucose uptake, reduces significantly, in HepG2 and SK-HEP-1 cells (Fig. 3A). Furthermore, HG causes time dependent increase in β-catenin protein level in HepG2 cells (Fig. 3B). Additionally, no change in β-catenin level was detected in cells cultured in the media containing variable amounts of glutamine and L-glucose (Supplementary Figure 2E). All together these results indicate that changes in level of β-catenin protein in HCC cells are glucose specific.

An enhanced cellular level of β-catenin is associated with increase in its transcriptional activity^18^,^21^,^22^. We observed an increase in level of β-catenin protein in the nuclear extract of HepG2 cells cultured in HG as compared to levels in the nuclear extracts of cells in NG or in HG treated with CytoB (Fig. 3C). Also, confocal microcopy analysis indicates enhancement in the levels of β-catenin protein in cells in HG as compared to cells in NG or in HG with CytoB (Fig. 3D). Elevated β-catenin protein level in cells cultured in HG further correlates with increase in the TOP/FOP ratio as compared to the ratio in NG. Also, Significant reduction in TOP/FOP ratio was observed in cells in HG treated with CytoB than in HG alone (Fig. 3E). These results collectively indicate an activation of canonical Wnt signaling pathway by β-catenin in HG conditions. Also, no change in pJNK level was detected in response to variable glucose culture conditions, indicating that high glucose does not activate non-canonical Wnt signaling pathway (Fig. 3F).

### High glucose induced proliferation of HCC cells is β-catenin dependent

**Figure 4:**
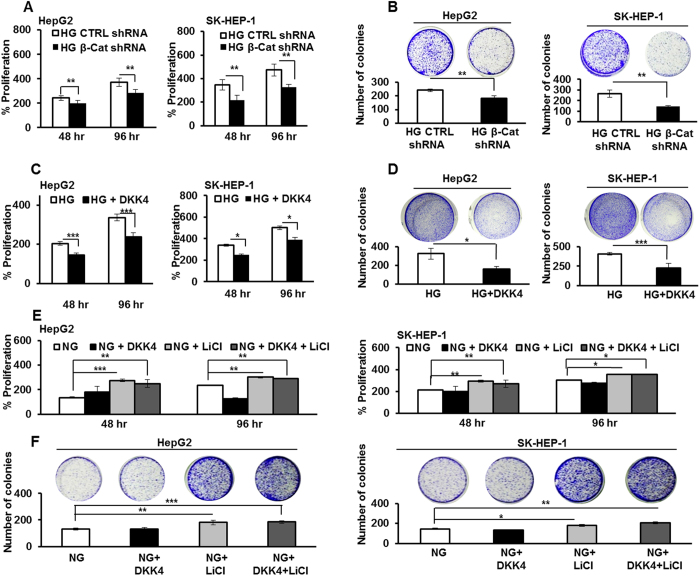
Glucose induced proliferation is dependent on β-catenin expression. (**A**) HepG2 and SK-HEP-1 cells were cultured in HG and transfected with β-catenin shRNA or control shRNA and percentage proliferation was determined by MTT assay. (**B**) HCC cells were transfected with β-catenin shRNA or control shRNA. Post 48 hr of transfection cells were cultured for additional 21 days. Thereafter, colonies were visualized by crystal violet stain and counted. (**C**) HCC cells were cultured in HG and HG + DKK4 protein in culture medium for 48 hr and 96 hr. MTT assay was performed and percentage proliferation was determined. (**D**) HCC cells were cultured in HG and HG + DKK4 recombinant protein in culture medium for 48 hr and cells were cultured for additional 21 days. Thereafter, colonies were visualized by crystal violet stain and counted. (**E**) HepG2 and SK-HEP-1 cells were cultured in NG, NG + LiCl, NG + DKK4 and NG + LiCl + DKK4 protein, for 48 hr and 96 hr. Percentage proliferation was determined by MTT assay. (**F**) Colony formation assay in HCC cells cultured in NG, NG + LiCl or NG + DKK4 or NG + LiCl + DKK4 protein, for 48 hr and cells were cultured for additional 21 days. Thereafter, colonies were visualized by crystal violet stain and counted. All the bar graphs represent the mean ± SD of an experiment done in triplicate (*P < 0.05, **P < 0.001, ***P < 0.0001).

Having demonstrated glucose dependent changes in β-catenin level as well as activity, next, we explored the role of β–catenin in glucose dependent increase in proliferation of HCC cells. To do that, cells were transfected with shRNA against β-catenin and after 24 hr they were cultured in HG conditions for 48 hr and 96 hr. MTT assay was performed to determine percentage change in cell proliferation. The proliferation of HepG2 and SK-HEP-1 cells in HG was significantly reduced upon transfection with β-catenin shRNA as compared to control shRNA (P < 0.005 each) (Fig. 4A). Also, in HepG2 and SK-HEP-1 cells cultured in HG and transfected with β-catenin specific shRNA, significantly less number of colonies were detected, than in cells transfected with control shRNA (P < 0.005 each) (Fig. 4B). Further, treatment of HCC cells cultured in HG with DKK4 protein significantly decreases cell proliferation and lesser number of colonies were detected by colony formation assay (Fig. 4C,D). These results indicate that DKK4 affects HG induced β-catenin dependent proliferation of HCC.

### Stabilization of β-catenin reverses the proliferation suppressive effect of DKK4 in HCC cells cultured in NG

**Figure 5:**
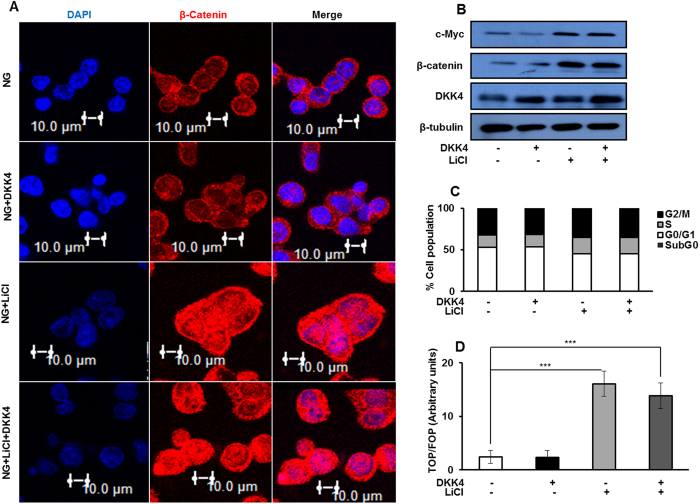
β-catenin stabilization in NG reverses effect of DKK4. (**A**) Immunofluorosence based confocal imaging of β–catenin protein in HepG2 cells cultured under indicated conditions for 16 hr. Bars represent 10 μm. (**B**) Immunoblotting for DKK4, β-catenin and c-Myc in HepG2 cells cultured in NG, NG + LiCl, NG + DKK4 and NG + LiCl + DKK4 protein, for 16 hr. Cropped blots are used in the main figure and full length blots are included in Supplementary Figure 9. (**C**) Cell cycle profile of HepG2 cells cultured in NG, NG + LiCl, NG + DKK4 and NG + LiCl + DKK4 protein, for 16 hr. Bar graphs represent percentage of cells in different phases of cell cycle by flow cytometry. (**D**) TCF reporter activity assay in HepG2 cells cultured in NG, NG + LiCl, NG + DKK4 and NG + LiCl + DKK4, protein for 16 hr. The luciferase intensities were normalized with Renilla intensities and data is represented as ratio of TOP/FOP. Bar graphs represent mean ± SE of three independent experiments (*P < 0.05, **P < 0.001, ***P < 0.0001).

So far, we observed that under NG culture condition, DKK4 is constitutively expressed which diminishes β-catenin induced cell proliferation. We therefore, investigated whether stabilization of β-catenin in NG by LiCl, which primarily acts by inhibiting GSK3β inside the cell^34^, interferes with the proliferation inhibitory effect of DKK4 and allows activation of canonical Wnt signaling. No changes were detected in GSK3β protein level in response to glucose (Supplementary Figure 2F). However, HCC cells in NG subjected to LiCl treatment proliferate rapidly than cells in NG alone, as determined by MTT assay (Fig. 4E). Also, more number of colonies were detected upon LiCl treatment in NG cells, and the number of colonies did not decrease upon treatment with DKK4 protein (Fig. 4F). Moreover, treatment of cells with LiCl causes an increase in β-catenin protein level in HepG2 cells cultured in NG, which was not reduced, even upon DKK4 protein treatment (Fig. 5A). Upon LiCl treatment, the level of DKK4 protein in whole cell lysate remains unchanged, whereas the level of c-Myc protein was increased under NG condition (Fig. 5B). This correlates with increase in percentage of cells in S-phase of cell cycle under NG cultured conditions, in presence of LiCl (Fig. 5C). TOP/FOP ratio was also significantly enhanced in LiCl treated HepG2 cells in NG together with DKK4 protein, than in NG alone (Fig. 5D). Furthermore, upon treatment of HepG2 cells cultured in NG with 6-Bromoindirubin-3′-oxime (BIO), a specific inhibitor of GSK3β, cell proliferate rapidly, which parallels with increase in β-catenin protein level and transcriptional activity, as indicated by elevated TOP/FOP ratio (Supplementary Figure 4). Collectively, these results indicate that DKK4 induced suppression of proliferation in NG can be abrogated by stabilizing β–catenin through inhibition of GSK3β either by LiCl or BIO.

### Hyperglycemia enhances the progression of HCC xenograft tumor

**Figure 6:**
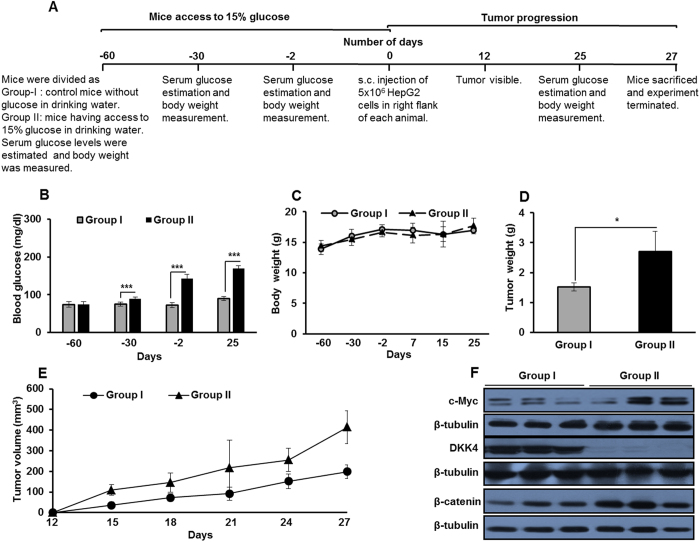
High glucose enhances HepG2 xenograft tumor growth *in vivo.* (**A**) Experimental layout for studying the effects of hyperglycemia on initiation and progression of HCC. (**B**) Blood glucose (mg/dl). (**C**) Body weight (g). (**D**) Tumor weight (g). (**E**) HepG2 cells (5 × 10^6^/mice) were injected s.c. on the right flank of each mouse. Tumor initiation and progression in Group-I and Group-II mice were recorded for 27 days. Data is represented as mean of five mice ± SD (*P < 0.05, **P < 0.001, ***P < 0.0001). (**F**) Protein level of c-Myc, β–catenin and DKK4 in representative three tumor samples each from mice of Group-I and Group-II were detected by western blotting. Cropped blots are used in the main figure and full length blots are included in Supplementary Figure 9.

Up till now, we demonstrated that high glucose causes proliferation of HCC cells *in vitro*. Further, these findings were evaluated in *in vivo*, and we investigated the effect of hyperglycemia on the progression of HCC xenograft tumors. NOD/SCID mice were grouped as Group I and Group II. Group II animals had access to 15% glucose in drinking water, as per the experimental layout (Fig. 6A). After 58 days, glucose levels in blood increased in mice supplemented with 15% glucose (Group-II) as compared to control mice (Group-I) (P < 0.001) (Fig. 6B). No significant difference in the body weight was detected in mice from both the groups (Fig. 6C). Following two months of glucose supplementation, equal numbers of HepG2 cells were subcutaneously injected into the right flank of each animal and these mice were observed for initiation and progression of tumors. Tumors progressed rapidly and tumor dimensions in glucose supplemented mice were at least twice the size of tumors in control fed animals on day 27 (Fig. 6E). Tumor weight was significantly higher in Group-II animals than in Group-I (Fig. 6D). In the lysate of tumors from Group-II mice, the levels of c-Myc and β-catenin proteins were more as compared to levels in the tumors from Group-I. Interestingly, DKK4 protein was barely detectable in lysates of tumors from glucose supplemented mice whereas in tumors from control group, DKK4 protein was abundantly present (Fig. 6F). These results suggest that glucose rich environment enhances progression of HCC tumor *in vivo.* In rapidly growing tumors DKK4 level is diminished whereas β-catenin level increases which are in concurrence with our *in vitro* findings.

### DKK4 knock down increases proliferation of HCC cells in NG

**Figure 7:**
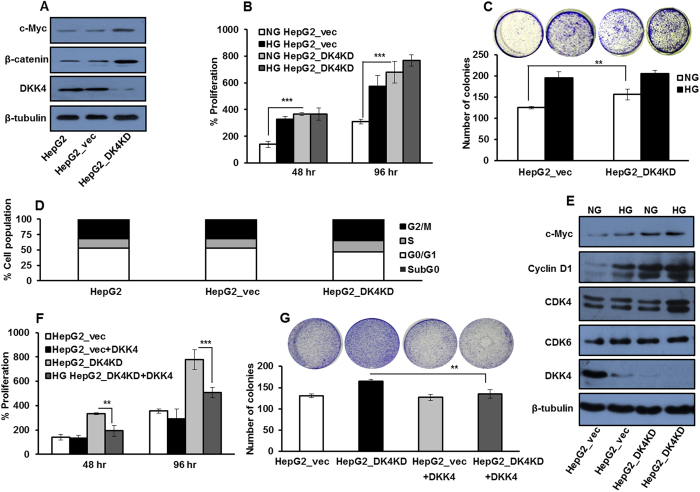
DKK4 affects glucose induced proliferation in HCC. (**A**) HepG2, HepG2_vec and HepG2_DK4KD cells were cultured in NG for 16 hr and western blotting was performed to detect protein levels of c-Myc, DKK4 and β–catenin. (**B**) HepG2_vec and HepG2_D4KD cells were cultured in NG and HG for 48 hr and 96 hr. Percentage cell proliferation was determined by MTT assay. (**C**) HepG2_vec and HepG2_D4KD cells were cultured in NG and HG and colonies were visualized by crystal violet stain and counted after 21 days. All the bar graphs represent the mean ± SD of an experiment done in triplicate (*P < 0.05, **P < 0.001, ***P < 0.0001). (**D**) Cell cycle profile of HepG2_vec and HepG2_D4KD cells cultured in NG for 16 hr. Bar graph represents percentage of cells in different phases of cell cycle by flow cytometry. (**E**) HepG2_vec and HepG2_D4KD cells were cultured in NG and HG for 16 hr and whole cell lysates were subjected to detection of c-Myc, Cyclin D1, CDK6, CDK4 and DKK4 proteins by western blotting. (**F**) HepG2_D4KD and HepG2_vec cells were cultured in NG and NG + DKK4 protein, for 48 hr and 96 hr. Thereafter, percentage cell proliferation was determined by MTT assay. (**G**) HepG2_D4KD and HepG2_vec cells were cultured in NG and NG + DKK4 protein. Colonies were visualized by crystal violet stain and counted after 21 days. All the bar graphs represent the mean ± SD of an experiment done in triplicate (*P < 0.05, **P < 0.001, ***P < 0.0001). Cropped blots are used in the main figure and full length blots are included in Supplementary Figure 10.

**Figure 8:**
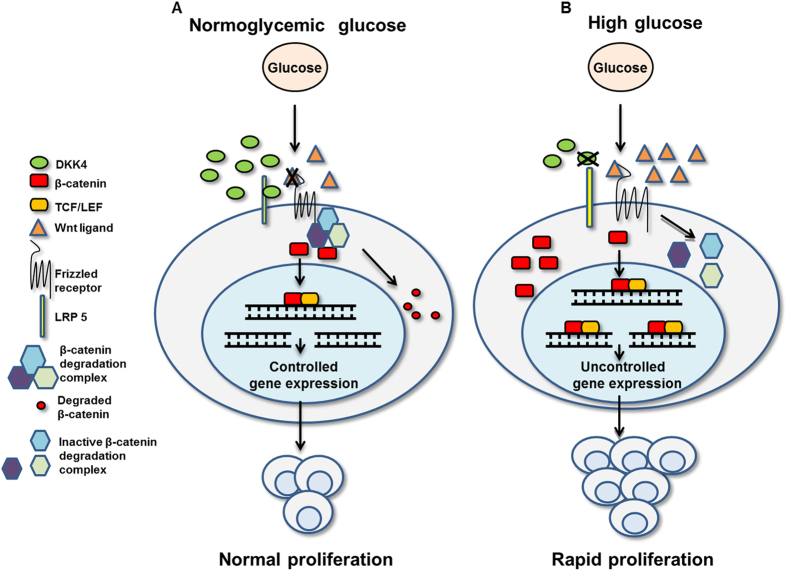
Schematic representation of glucose induced regulation of DKK4 and effect over HCC proliferation. (**A**) Normoglycemic glucose promotes sustained expression of DKK4 protein. DKK4 antagonizes activation of canonical Wnt signaling by facilitating degradation of β–catenin in cytosol and thus reducing its transcriptional activation thereby causing decrease c-Myc level. Increased DKK4 expression affects progression of cells at S-phase of cell cycle and therefore limits proliferation of HCC cells. (**B**) High glucose diminishes DKK4 expression allowing activation of canonical Wnt signaling because of inactivation of β–catenin degradation complex, by Wnt3a proteins. Increase in β–catenin level enhances its transcriptional activity and promotes c-Myc expression which causes uncontrolled proliferation of HCC cells.

To examine the specificity of DKK4 in glucose induced proliferation of HCC cells, we developed HepG2 cell line which constitutively expresses shRNA against DKK4 transcript (HepG2_DK4KD) and a cell line expressing control shRNA (HepG2_vec). The developed cell lines were validated for DKK4 knockdown, c-Myc and β–catenin protein levels by immunoblotting (Fig. 7A). DKK4 knockdown increases proliferation in HepG2 cells cultured in NG for 48 hr and 96 hr as compared to control shRNA transfected cells (P < 0.001 each), as assessed by MTT assay (Fig. 7B). Also, more number of colonies are detected in HepG2_DK4KD cells as compared to HepG2_vec cells cultured in NG conditions (Fig. 7C). In DKK4 knockdown cells, increase in percentage of cells in S-phase of the cell cycle under NG was detected (Fig. 7D). Parallely, the levels of cell cycle regulatory proteins (c-Myc, Cyclin D1, CDK4 and CDK6) were elevated in DKK4 knockdown cells in NG and these levels were comparable to levels in cells cultured in HG (Fig. 7E). Supplementation of DKK4 protein in HepG2_DK4KD cells causes suppression of glucose promoted growth as determined by MTT and colony formation assays (Fig. 7F,G). Our data suggest a proliferation suppressive role of DKK4 by specifically inhibiting the progression of cell cycle events from G0/G1 to S phase. A schematic representation of our hypothesis and results obtained is depicted in Fig. 8.

## Discussion

The most important characteristic property of cancer cells is their ability to rapidly uptake glucose and utilizing it efficiently, offering them a selective proliferative advantage. Further, this property of cancer cells might be amplified during transient hyperglycemic environment or under chronic hyperglycemic state as in case of pre-existing diabetes. It has been reported that hyperglycemic insult is associated with increased risk of HCC^35^,^36^. Also, cancer progresses rapidly in glucose and fructose promoted hyperglycemia^37^. However, the mechanism underpinning these important findings remain unclear. In this study, we attempted to elucidate the molecular mechanism and alterations in the signaling cascades which may be contributing towards rapid proliferation of HCC in glucose rich environment.

Our *in vitro* and *in vivo* data demonstrated that glucose enriched condition specifically increases the levels of c-Myc and β–catenin in HCC. Also, our results suggest that glucose uptake and utilization by HCC cells is associated with change in cell cycle progression because of alterations in expression levels of proteins specific to the regulation of G_0_/G_1_/S phases. Dang, C.V. and Wang, H. *et al.* have reported that elevated level of c-Myc is an important molecular event which promotes proliferation by specifically enhancing recruitment of cells to the S-phase of cell cycle^38^,^39^. Also, few studies have highlighted the role of glucose in the induction of c-Myc dependent oncogenic transformation^22^,^25^,^27^,^40^. Additionally, it has been reported that in higher grades of human HCC biopsy samples c-Myc protein levels are elevated^41^,^42^. Change in the expression of c-Myc protein is regulated by mitogenic signals such as Wnt proteins, EGF, TGF, etc.,^43^. In HCC tumors, c-Myc expression is primarily regulated by activation of canonical Wnt signaling because of nuclear retention of β-catenin^44^ and is involved in proliferation, evasion, metastasis and poor differentiation^45^. However, emerging evidences document that Wnt mediated stimulation alone is not enough to promote the nuclear accumulation of β-catenin and hyperglycemic environment is also contributing to canonical Wnt signaling in a variety of human cancer cells^25^. In this direction, our results indicate that high glucose selectively activates canonical Wnt signaling, which enhances the transcription of β-catenin responsive genes in HCC. Also, these evidences direct the attention towards upstream factors which might have direct involvement in differential regulation of the canonical Wnt signaling pathway under variable glucose concentrations.

Sato, H. *et al.* have reported that canonical Wnt signaling is regulated by a dynamic balance between Wnt ligands and Wnt antagonists like DKK family of secretory proteins^31^. Also, Fatima, S. *et al.* have shown that among various DKK proteins, DKK4, a tumor suppressor, is down regulated in 67.5% of HCC tumors and it has an inverse relationship with β-catenin protein stabilization in cytosol^32^. In accordance with this, we observed that high glucose specifically reduces the expression of DKK4 with concomitant increase in Wnt3a and β–catenin protein levels. Furthermore, the relationship between glucose and DKK4 became apparent because stabilization of β-catenin by inhibiting GSK3β under normoglycemic condition causes abrogation of DKK4 functionality thereby facilitating cellular proliferation. Critical role of DKK4 in regulating cell cycle events was confirmed as shRNA mediated knockdown of DKK4 unlocks growth suppression under normoglycemia.

Further, in limited HCC biopsy samples derived from patients with and without pre-existing diabetes, we detected differential immunostaining pattern for DKK4 and β–catenin proteins (Supplementary Figure 5 and Supplementary table) which point towards possible existence of a link between high glucose and modulation of proliferative signaling cascades. These observations need to be ascertained in large sample size. Previously, it has been demonstrated that DKK4 expression is significantly reduced in aggressive HCC tumors than in adjacent normal liver tissues^32^ and in, specific subsets of HCC tissues with reduced thyroid hormone receptor^46^. Therefore, it is likely that gradual decrease in the functionality of DKK4 might be an early event in hepatocarcinogenesis which is further hastened by underlying hyperglycemic status. Though, the correlation between DKK4 and HCC tumors is noticeable, further analysis by large scale screening of biopsy samples will strengthen our findings. Recently, role of various DKKs in hepatocellular carcinoma has been reviewed and DKK4 down regulation has been proposed to be a signature event in HCC development^29^,^47^ which is also evident in TCGA cohort based analysis.

Further, we explored the mechanism involved in regulation of DKK4 gene in response to glucose and observed that acetylation specific epigenetic modifications are involved in regulation, in response to physiological concentrations of glucose (Data unpublished). These findings are in accordance with a previous study in which it has been reported that DKK4 is regulated by acetylation specific response^46^. So far, regulators of DKK4 have not been studied in detail. Therefore, further investigations are required to determine the upstream chromatin modulators involved in glucose specific alteration of DKK4 in HCC.

In summary, we have shown for the first time that high glucose facilitated HCC proliferation is a consequence of the expressional and functional interplay between DKK4, β-catenin and c-Myc. In HCC cells, under normoglycemic conditions, DKK4 antagonizes activation of canonical Wnt signaling and thus checks proliferation specifically at G0/G1/S phase of the cell cycle. Hyperglycemia diminishes DKK4 protein which causes activation of canonical Wnt signaling pathway through Wnt3a ligand mediated enhanced translocation of β–catenin into nucleus, thereby promoting proliferation of HCC cells.

## Materials and Methods

### Chemicals and reagents

Methylthiazoletetrazolium (MTT), lithium chloride (LiCl), cytochalasin B (CytoB), L-glucose (L-Glu), glutamine (Glt), D-glucose and mannitol (Mntl) were purchased from Sigma-Aldrich (Sigma Aldrich, MO, USA). 6-Bromoindirubin-3′-oxime (BIO) was purchased from Merck Millipore (Merck Millipore, MA, USA). Antibodies against DKK4 (sc-25519), GLUT-1 (sc-7903), GLUT-2 (sc-9117), Cyclin D1(sc-717), CDK4 (sc-260), CDK6 (sc-7181), c-Myc (sc-764), Wnt3a (sc-28824), pJNK (sc-6254), JNK (sc-474), pMEK (sc-7995), MEK (sc-13069), Raf (sc-227), pERK (sc-7383), ERK (sc-154), pAkt (sc-7985), AKT (sc-8312), GSK3β (sc-9166), β-tubulin (sc-9104) and Histone H1 (sc-10806), HRP-conjugated (sc-2004), (sc-2031), (sc-2033), FITC conjugated (sc-3839) and Rhodamine conjugated (sc-358922) antibodies were purchased from Santa Cruz Biotechnology (Santa Cruz Biotechnology, CA, USA). Antibody for β-catenin (610154) was purchased from BD Bioscience (BD Bioscience, NA, USA). Antibody for pGSK3β (9336S) was purchased from Cell Signaling technologies (Cell Signaling technologies, MA, USA). Wnt3a (5036-WN) and DKK4 (1269-DK) recombinant proteins were purchased from R&D Systems (R&D Systems, MN, USA). For developing stable cell lines, shRNA for DKK4 and control plasmids were purchased form Open Biosystems (Thermo Fisher Scientific, PA, USA).

### Human HCC clinical tissues

Experiments done with all human tissues were performed according to the Institutional guidelines, following protocols approved by the Institutional Ethic Committee (IEC) of National Centre for Cell Science (NCCS), Pune, India and Institutional Ethic Committee of Sri Dharmasthala Manjunatheshwara Medical Sciences and Hospital, Dharwad, Karnataka, India. We confirm that “Informed consent” was obtained from all subjects and submitted to institutional ethical committee of Sri Dharmasthala Manjunatheshwara Medical Sciences and Hospital, Dharwad, Karnataka, India. A total of seven trucut HCC biopsy samples were obtained from patients at Sri Dharmasthala Manjunatheshwara Medical Sciences and Hospital during 2014–2015. The following clinical pathological data were noted: age, gender, tumor grade, presence of HBV/C, diabetes, NAFLD and liver cirrhosis. Tissue samples were fixed in paraffin blocks immediately after resection and further processed for immunohistochemical studies.

### Hyperglycemia and *in vivo* tumor growth experiment

All the animal experiments were performed according to the Institutional guidelines, following protocols approved by the Institutional Animal Ethic Committee (IAEC) of National Centre for Cell Science (NCCS), Pune, India. Five to six weeks old male NOD/SCID mice (weight 15 ± 2 g) were acquired from experimental animal facility (EAF) of National Centre for Cell Science, Pune, India. Mice were randomly divided into two groups (n = 5). Group-I mice were allowed free access to normal drinking water whereas Group–II mice were allowed free access to water supplemented with 15% glucose throughout the experiment. At regular intervals body weight was monitored and glucose was estimated by using rapid glucose analyzer (Accu-Chek Sensor Comfort, Roche Diagnostics, Germany) in the blood collected by an approved tail cap method.

When the blood glucose levels reached above 130 mg/dl, mice were considered hyperglycemic. At day 60, HepG2 cells (5 × 10^6^/mice) were injected subcutaneously (s.c.) on the right flank of each mouse. After 12–18 days, palpable tumor appeared. The size of tumors during the course of the experiment was measured using Vernier caliper, in two dimensions. Tumor volume (mm) was calculated according to the formula (A^2^ × B) × 0.52 (A, length; B, width; all parameters in millimeters). At the end of experiment, mice were sacrificed by cervical dislocation and tumors were excised and tumor weight was measured. Tumor lysates were prepared and used for western blotting.

### Cell culture

Human hepatocellular carcinoma cells (HepG2, SK-HEP-1, Chang Liver and WRL 68) were purchased from ATCC and maintained in our in house repository at National Centre for Cell Science, Pune, India. All the cells were grown in Dulbecco modified eagles medium (DMEM) either in normoglycemic glucose, 5.5 mM (NG) or in high glucose, 25 mM (HG), depending upon the experiments and supplemented with 10% heat inactivated fetal bovine serum (Hyclone, UT, USA), penicillin (100 U/ml) and streptomycin 100 μg/ml (Invitrogen Life Technologies, CA, USA), at 37 °C in an incubator in the presence of 5% CO_2_ (Thermo Scientific, NC, USA).

### Inhibitor treatment

HCC cells were plated and cultured for 24 hr at 37 °C. Thereafter, cells were starved of serum and glucose for 2 hr. Fresh DMEM containing 10% FBS with NG or HG along with Mntl (19.5 mM), CytoB (10 μM), LiCl (20 mM), DKK4 (200 nM) and BIO (1μM), was added as per the experimental plan. Cells were cultured for additional 16 hr or 48 hr or 96 hr depending upon the experiment. Thereafter, cells were processed for MTT assay, Colony formation assay, Glucose utilization assay, Indirect ELISA, Immunofluorescence or Cell cycle analysis or for lysate preparation for western blotting, Glucose uptake assay, Dual luciferase assay and RNA extraction, as mentioned below in individual sections.

### Cell proliferation assay

HCC cells (2 × 10^3^) were plated in 96 well plates and allowed to adhere for 24 hr at 37 °C. Next day, cells were starved of serum and glucose for 2 hr. Thereafter, fresh DMEM containing 10% FBS with NG, HG, NG + Mntl, HG + CytoB, NG + LiCl, NG + DKK4, HG + DKK4, NG + LiCl + DKK4 and NG + BIO, was added and cells were cultured for 48 and 96 hr. Percentage cell proliferation was assessed by MTT assay and represented by bar graphs as mean ± SD, as described previously^48^.

### Colony formation assay

HCC cells (5 × 10^2^) were plated in 12 well plates and allowed to adhere for 24 hr at 37 °C. Next day, cells were starved of serum and glucose for 2 hr. Thereafter, fresh DMEM containing 10% FBS with NG, HG, NG + Mntl, HG + CytoB, NG + LiCl, NG + DKK4, HG + DKK4 and NG + LiCl + DKK4, was added and cells were cultured for further 48 hr. Subsequently, medium was replaced with NG or HG medium and cells were incubated for an additional 21 days at 37 °C in CO_2_ incubator with medium change on every 2–3 day. Cells were then fixed (3% paraformaldehyde and 0.02% glutaraldehyde in PBS) and stained with 0.05% crystal violet. Colonies having cells more than 50 cells were counted by vertical microscope (Olympus, Tokyo, Japan) and plotted in bar graphs as mean + SD.

### Cell cycle analysis

HepG2 cells (3 × 10^5^) were plated in 35 mm plates and allowed to adhere for 24 hr at 37 °C. Next day, cells were starved of serum and glucose for 2 hr. Thereafter, fresh DMEM containing 10% FBS with NG, HG, NG + Mntl, HG + CytoB, NG + LiCl, NG + DKK4, HG + DKK4 and NG + LiCl + DKK4, was added and cells were cultured for 16 hr. Thereafter, cells were collected and processed for cell cycle analysis as described elsewhere^49^. Data was analyzed using CellQuest Pro software (BD Biosciences, CA, and USA) and bar graphs represent percentage of cells in different phases of cell cycle by flow cytometry of an experiment done in triplicate.

### Cell lysis preparation and immunoblotting

HCC cells (3 × 10^5^) were plated in 35 mm culture plates and allowed to adhere for 24 hr at 37 °C. Next day, cells were starved of serum and glucose for 2 hr. Thereafter, fresh DMEM containing 10% FBS with NG, HG, NG + Mntl, HG + CytoB, NG + LiCl, NG + DKK4, HG + DKK4, NG + LiCl + DKK4, NG + BIO and NG + BIO + DKK4, was added and cells were cultured for 16 hr. Thereafter, whole cell lysates or nuclear and cytosolic extracts were prepared and immunoblotting was performed as described previously^48^. For experiments in which time intervals are variable, HepG2 cells were plated as mentioned above and after serum and glucose starvation for 2 hr cells were cultured in NG or HG media for 0, 3, 6, 12 or 24 hr. Thereafter, cell lyates were prepared and processed for western blotting as described above.

### Glucose uptake assay

HepG2 cells (3 × 10^3^) were plated in 12 well tissue culture plates and allowed to adhere for 24 hr at 37 °C. Next day, cells were starved of serum and glucose for 2 hr. Thereafter, fresh DMEM containing 10% FBS with NG, HG, NG + Mntl and HG + CytoB was added and cells were cultured for 16 hr. Glucose uptake assay was performed as mentioned earlier^50^. Radioactive glucose uptake was measured and represented as counts per million (CPM) in scintillation counter (Packard, MN, U.S.A.). For experiments in which time intervals are variable, HepG2 cells were plated as mentioned above and after serum and glucose starvation for 2 hr cells were cultured in NG or HG for 0, 3, 6, 12 or 24 hr. Thereafter, radioactive glucose uptake was measured as described above.

### Glucose utilization assay

HepG2 cells (3 × 10^3^) were plated in 12 well tissue culture plates and allowed to adhere for 24 hr at 37 °C. Next day, cells were starved of serum and glucose for 2 hr. Thereafter, fresh DMEM containing 10% FBS with NG, HG, NG + Mntl and HG + CytoB was added and cells were cultured for 16 hr. For measuring glucose utilization, residual glucose present in the culture medium was measured using rapid glucose analyzer (Accu-Chek Sensor Comfort, Roche Diagnostics, Germany) according to manufacturer’s instructions. Consumed glucose was estimated by subtracting the remaining residual glucose in the medium from the initial concentration in control medium (450 mg/dl). The experiments were performed at least in triplicate and values were normalized to total number of cells. For experiments in which time intervals are variable, HepG2 cells were plated as mentioned above and after serum and glucose starvation for 2 hr cells were cultured in NG or HG for 0, 3, 6, 12 or 24 hr. Thereafter, glucose utilization was measured as described above.

### Dual luciferase assay

Dual luciferase assay for determining TCF reporter activity, which is indicative of transcriptional activity of β–catenin, was performed as described previously^51^. Briefly, HepG2 cells (2 × 10^3^) were plated in 48 well culture plates for 24 hr at 37 °C. Cells were transfected with TCF reporter plasmid (16x-TOPFlash) or with reporter plasmid containing mutant TCF binding site (16x-FOPFlash) (kind gift from Dr. Randall T. Moon, University of Washington, Washington, USA) along with transfection control Renilla luciferase reporter vector, pRL-TK (Promega, WI, USA) using Lipofectamine 2000 (Invitrogen Life Technologies, CA, USA). After 48 hr of transfection, cells were starved of serum and glucose for 2 hr. Thereafter, fresh DMEM containing 10% FBS with NG, HG, NG + Mntl, HG + CytoB, NG + LiCl, NG + DKK4, HG + DKK4, NG + LiCl + DKK4, NG + BIO and NG + BIO + DKK4, was added and cells were cultured for 16 hr. Cells were collected, suspended in reporter lysis buffer and luciferase assay was performed using dual luciferase assay system (Promega, WI, USA) in the GloMax Multi Detection system (Promega, WI, USA). The luciferase intensities were normalized with Renilla intensities and represented as ratio of TOP/FOP in bar graphs.

### RNA extraction, cDNA synthesis and quantitative RT-PCR

HCC cells (3 × 10^5^) were plated in 35 mm culture plates and allowed to adhere for 24 hr at 37 °C. Next day, cells were starved of serum and glucose for 2 hr. Thereafter, fresh DMEM containing 10% FBS with NG, HG and HG + CytoB was added and cells were cultured for 16 hr. Total RNA from the cells was extracted using TRIzol reagent (Invitrogen Life Technologies, CA, USA) as per the manufacturer’s instructions. cDNA synthesis and semi quantitative PCR was performed as described previously^52^ with following primers ; DKK1 5′-CTTTCCCCTCTTGAGTCCTTCTG-3′ (F), 5′-CATAGCGTGACGCATGCAGCGTT-4′ (R); DKK2 5′-GCAGTGATAAGGAGTGTGAAGTT-3′ (F), 5′-AATGCAGTCTGATGATCGTAGGC-3′ (R); DKK3 5′-CTGGGAGCTAGAGCCTGAT-3′ (F), 5′-TCATACTCATCGGGGACCTC-3′ (R); and β-actin 5′-ATCTGGCACCACACCTTCTACAATGAGCTGCG-3′ (F), CGTCATACTCCTGCTTGCTGATCCACATCTGC-3′ (R) at annealing temperature of 59 °C for 25–30 cycles.

Quantitative PCR was performed using SYBR Green PCR Master Mix (Invitrogen Life Technologies, CA, USA) in Rotor-Gene Q (Qiagen, CA, USA) utilizing following pairs of primers for DKK4 5′-GCAGGACGATATCTCTAGCT-3′ (F), 5′-GTCTCCAACTTATCTCCTCCATTC-3;(R), for β–catenin 5′-GGTGAAAATGCTTGGTTCACC -3′ (F) and 5′-CGCACTGCCATTTTAGCTCC-3′ (R) and for β-Actin 5′-GGTGAAAATGCTTGGTTCACC-3′ (F),5′-CGCACTGCCATTTTAGCTCC-3′ (R). The reactions were performed under following cycle conditions: 50 °C for 2 min, 95 °C for 10 min, 40 cycles of 95 °C for 15 Sec and 60 °C for 1 min, in triplicate. The relative mRNA expression was normalized with β–actin as an internal control. 2-(delta) (delta) Ct method was used to calculate the fold change in gene expression.

### shRNA transfection and establishment of DKK4 knock down cell line

shRNA against human β-catenin was generated by annealing the following oligos and cloning at BglII/XhoI sites of pSUPER vector (kind gift from Dr. Mary Dasso, NIH, USA). Human β-catenin sense oligo: 5′GATCCCCTCCCTGAACTGACAAAACTTTCAAGAGAAGTTTTGTCAG TTCAGGGATTTTTGGAAC3′, human β-catenin anti-sense oligo: 5′TCGAGTTCCAA AAATCCCTGAACTGACAAAACTTCTCTTGAAAGTTTTGTCAGTTCAGGGAGGG 3′. HepG2 cells, (2 × 10^5^) were plated in 35 mm culture dish and, (2 × 10^3^) cells in 96 well plate. Cells were transfected with 100 nM of shRNA specific for β-catenin or non-specific control shRNA (pSUPER vector) using Lipofectamine 2000 (Invitrogen Life Technologies, CA, USA). Eight hour post-transfection, the media was changed and cells were further incubated for 48 hr. Then cells were starved of serum and glucose for 2 hr. Thereafter, fresh DMEM containing 10% FBS with NG and HG was added and cells were cultured for 16 hr or 48 hr and 96 hr and then processed for MTT assay or cell cycle analysis or for lysate preparation for western blotting, as mentioned above.

For development of DKK4 knockdown cell line, HepG2 cells (2 × 10^5^) in 35 mm culture dish were transfected with either shRNA specific for DKK4 (RHS4531) or non-specific control shRNA (TLP4614) (Thermo Fisher Scientific, PA, USA) using Lipofectamine 2000 (Invitrogen Life Technologies, CA, USA). Transfected cells were grown in DMEM with 10% FBS and 500 μg/ml G418 antibiotic for 8 weeks. Thereafter, DKK4 knock down was confirmed by western blotting.

### Indirect ELISA

Secreted levels of Wnt3a and DKK4 proteins in media were detected by indirect ELISA. Briefly, 7 × 10^7^ HepG2 cells were plated in 60 mm cell culture dish and allowed to adhere for 24 hr at 37 °C. Next day, cells were starved of serum and glucose for 2 hr. Thereafter, fresh DMEM containing 10% FBS with NG, HG and HG + CytoB was added and cultured for 16 hr. Media was collected and lyophilized to half the volume in Speed Vac (Thermo Fisher Scientific, PA, USA). ELISA plates (Becton Dickenson, NJ, USA) were coated with 50 μl of above media and incubated at 37 °C for 3 hr. Blocking was done using 2% BSA in phosphate buffer saline (PBS) at 37 °C for 2 hr. After washing with PBS, coated antigens were probed with 50 μl of primary antibodies for Wnt3a and DKK4 (200 ng/50 μl) (Santa Cruz Biotechnology, CA, USA) for 3 hr at 37 °C. Following washing, samples were incubated with 50 μl of HRP-conjugated secondary antibody (200 ng/50 μl) (Santa Cruz Biotechnolgy, CA, USA) for 3 hr at 37 °C. Finally, ABTS (2, 2′-azinobis-3-ethylbenzothiazoline-6-sulfonic acid) (Sigma Aldrich, MO, USA) in 0.1 M Na-citrate buffer (pH 4.0) was added to each well and incubated for 10 min at room temperature before taking absorbance at 405 nm. Standard curves were prepared with different concentrations (1 pg to 100 ng) of DKK4 and Wnt3a recombinant proteins. Concentrations of these proteins in media is determined and represented in bar graphs as mean ± SD.

### Immunofluorescence

HepG2 cells (3 × 10^2^) were seeded on cover slips and allowed to adhere for 24 hr at 37 °C. Next day, cells were starved of serum and glucose for 2 hr. Subsequently, fresh DMEM containing 10% FBS with NG, HG, HG + CytoB, NG + LiCl, NG + DKK4, HG + DKK4 and NG + LiCl + DKK4, was added and cells were cultured for 16 hr. Thereafter, immunofluorescence analysis was performed as described previously^53^.

### Immunohistochemical staining

Fine sections (4 μm) were prepared from formalin fixed paraffin embedded tumor tissues and fixed on glass slides (Safeline Histopathology, Pune, India). For immunohistochemistry, slides were deparaffinized by xylene solution twice for 10 min and subsequently rehydrated in graded alcohol (100%, 95%, 70% and 50%). For antigen retrieval, slides were boiled in sodium citrate buffer (0.01 M, pH 5) at 100 °C for 10 min and allowed to cool at room temperature. BSA (0.2%) was used for blocking for 1 hr. After washing with TBST, slides were probed with DKK4 (1:100) (Santa Cruz Biotechnology, CA, USA) and IHC specific β–catenin (1:100) antibody (BD Bioscience, NA, USA) and incubated at 4 °C overnight. Slides were washed with TBST and probed with compatible FITC or TRITC conjugated secondary antibody for 3 hr. After five washes with TBST, tissue sections were layered with mounting medium containing DAPI (Santa Cruz Biotechnology, CA, USA). Tissues were examined and images were captured with a confocal microscope at 60x magnification (LSM510 or META, Carl Zeiss). Images were subsequently processed by LSM image analysis software (Carl Zeiss, IL, USA).

### Bioinformatics analysis

The ONCOMINE (www.oncomine.com, December 2015, Thermo Fisher Scientific, Ann Arbor, MI, USA) database was used for bioinformatics analysis. We have searched for differential gene expression in TCGA cohort using appropriate filtrating criteria. The criteria were: (i) cancer vs. normal to search for expression in liver cancer (ii) grade wise to search for expression in liver cancer. All datasets were filtered by threshold criteria by P value 1E-4, Fold change 2 and gene rank top 10%.

### Statistical analysis

Experiments were performed in triplicates or otherwise as mentioned. All data are presented as mean + standard deviation (SD) of replicates or as mean ± standard error (SE) as mentioned. Statistical comparison was performed by Student’s 2-tailed unpaired t-test by using Sigma Plot software (Systat Software Inc., CA, USA). The values of P < 0.05 were considered statistically significant.

## Additional Information

**How to cite this article**: Chouhan, S. *et al.* Glucose induced activation of canonical Wnt signaling pathway in hepatocellular carcinoma is regulated by DKK4. *Sci. Rep.* **6**, 27558; doi: 10.1038/srep27558 (2016).

## Supplementary Material

Supplementary Information
