# Supplementary material for: Glucose induced activation of canonical Wnt signaling pathway in hepatocellular carcinoma is regulated by DKK4
Source: Sci Rep. 2016 Jun 8;6:27558. doi: 10.1038/srep27558 (PMC4897783; doi:10.1038/srep27558)
Supplement: Supplementary Information [file srep27558-s1.doc]

**Glucose induced activation of canonical Wnt signaling pathway in hepatocellular carcinoma is regulated by DKK4**

Surbhi Chouhan^1^, Snahlata Singh^1^, Dipti Athavale^1^, Pranay Ramteke^1^, Vimal Pandey^1,2^, Jomon Joseph^1^, Rajashekar Mohan^3^, Praveen Kumar Shetty^3^ and Manoj Kumar Bhat^1*^

**
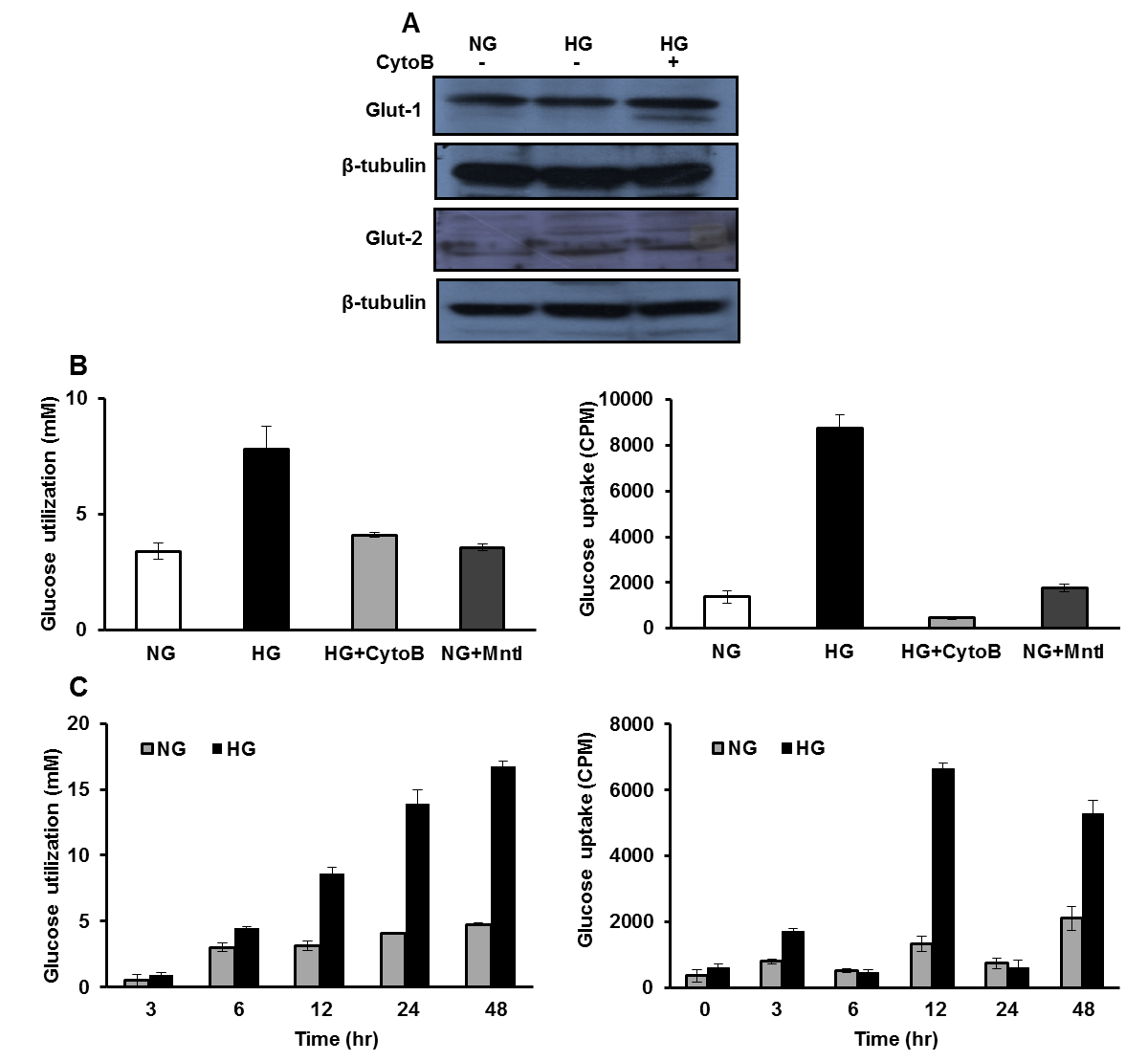
**

**Supplementary Figure 1. High glucose increases receptor mediated uptake in HCC cells.**

(a) HepG2 cells were cultured in NG, HG and HG+CytoB for 16 hr. Whole cell lysates were subjected to western blotting and levels of GLUT-1 and GLUT-2 proteins were detected.

(b) HepG2 cells were cultured in NG, NG+Mntl, HG and HG+CytoB for 16 hr. Glucose levels were quantified in culture media and glucose utilization is represented in mM (Left panel). Cells were further processed for glucose uptake assay and radiolabelled glucose uptake was measured as counts per million (CPM) (Right panel).

(c) HepG2 cells were cultured in NG, NG+Mntl, HG and HG+CytoB for indicated time intervals. Glucose levels were quantified in culture media and glucose utilization is represented in mM (Left panel). Cells were further processed for glucose uptake assay and radiolabelled glucose uptake was measured as counts per million (CPM) (Right panel). Bar graphs represent the mean±SD of an experiment done in triplicate.

**
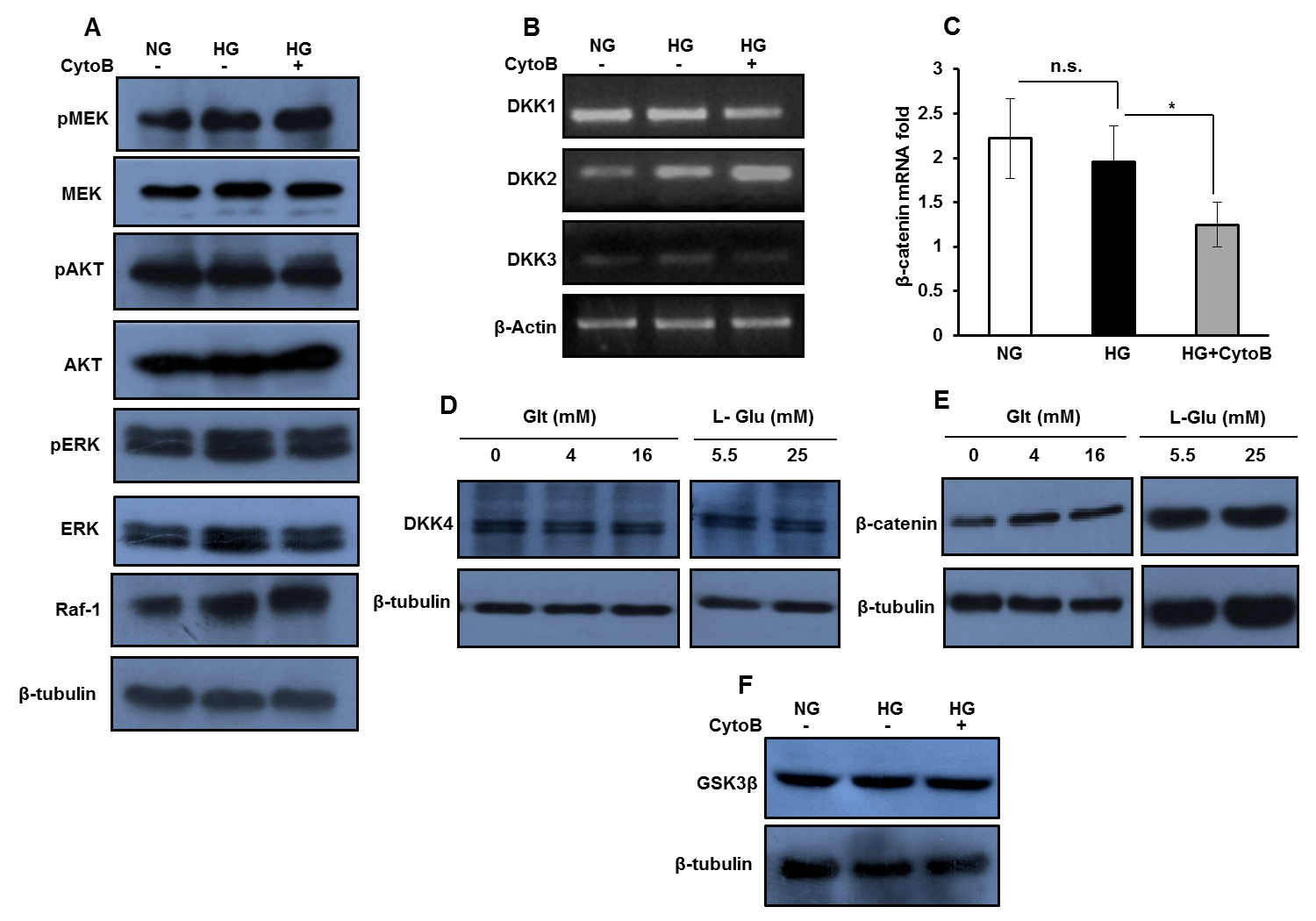
**

**Supplementary Figure 2. High glucose alters canonical Wnt signaling.**

(a) Western blotting analysis for proteins associated with proliferative pathways in lysates of HepG2 cells cultured in NG, HG and HG+CytoB for 16 hr.

(b) Semi quantitative PCR for detection of mRNA levels of DKK-1,-2 and -3 in HepG2 cells cultured in NG, HG and HG+CytoB for 16 hr.

(c) HepG2 cells were cultured in NG, HG and HG+CytoB for 16 hr. Total RNA was isolated and cDNA was prepared to determine relative mRNA fold expression of β-catenin by quantitative real time RT-PCR. Bar graphs represent the mean±SD of an experiment done in triplicate (*P<0.05), n.s. represents non significant P value (P>0.05).

(d) Immunoblotting for DKK4 in HepG2 cells cultured under variable glutamine (Glt) and L-glucose (L-Glu) culture conditions for 16 hr.

(e) Immunoblotting for β-catenin in HepG2 cells cultured under variable glutamine and L-glucose culture conditions for 16 hr.

(f) Western blotting analysis for pGSK3β and GSK3β proteins in lysates of HepG2 cells cultured in NG, HG and HG+CytoB for 16 hr.


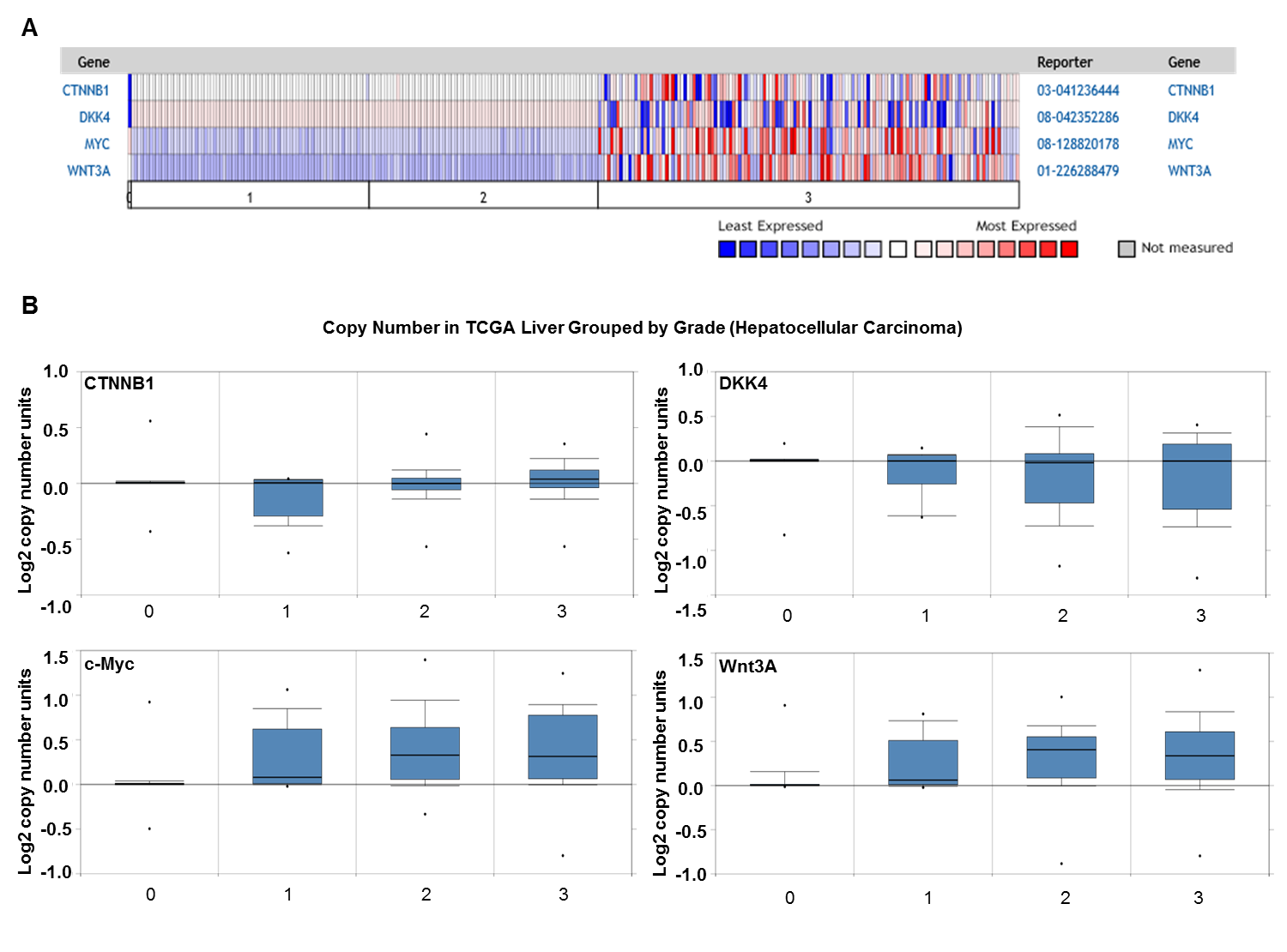


**Supplementary Figure 3. TCGA analysis of normal liver v/s HCC for expression and grade wise distribution of CTNNB1, DKK4, c-Myc and Wnt3a.**

(a) CTNNB1, DKK4, c-Myc and Wnt3a expression was evaluated in normal liver vs. cancer tissue from TCGA cohort obtained from ONCOMINE database. (0) no value (n=1), (1) blood (n=78), (2) liver (n=75) and (3) hepatocellular carcinoma (n=138).

(b) Grade wise expression of CTNNB1, DKK4, c-Myc and Wnt3a in HCC from TCGA cohort study obtained from ONCOMINE database. (0) no value (n=173), (1) grade 1 (n=14), (2) grade 2 (n=62) and (3) grade 3 (n=37). All datasets were filtered by threshold criteria by P value 1E-4, Fold change 2 and gene rank top10%.

**
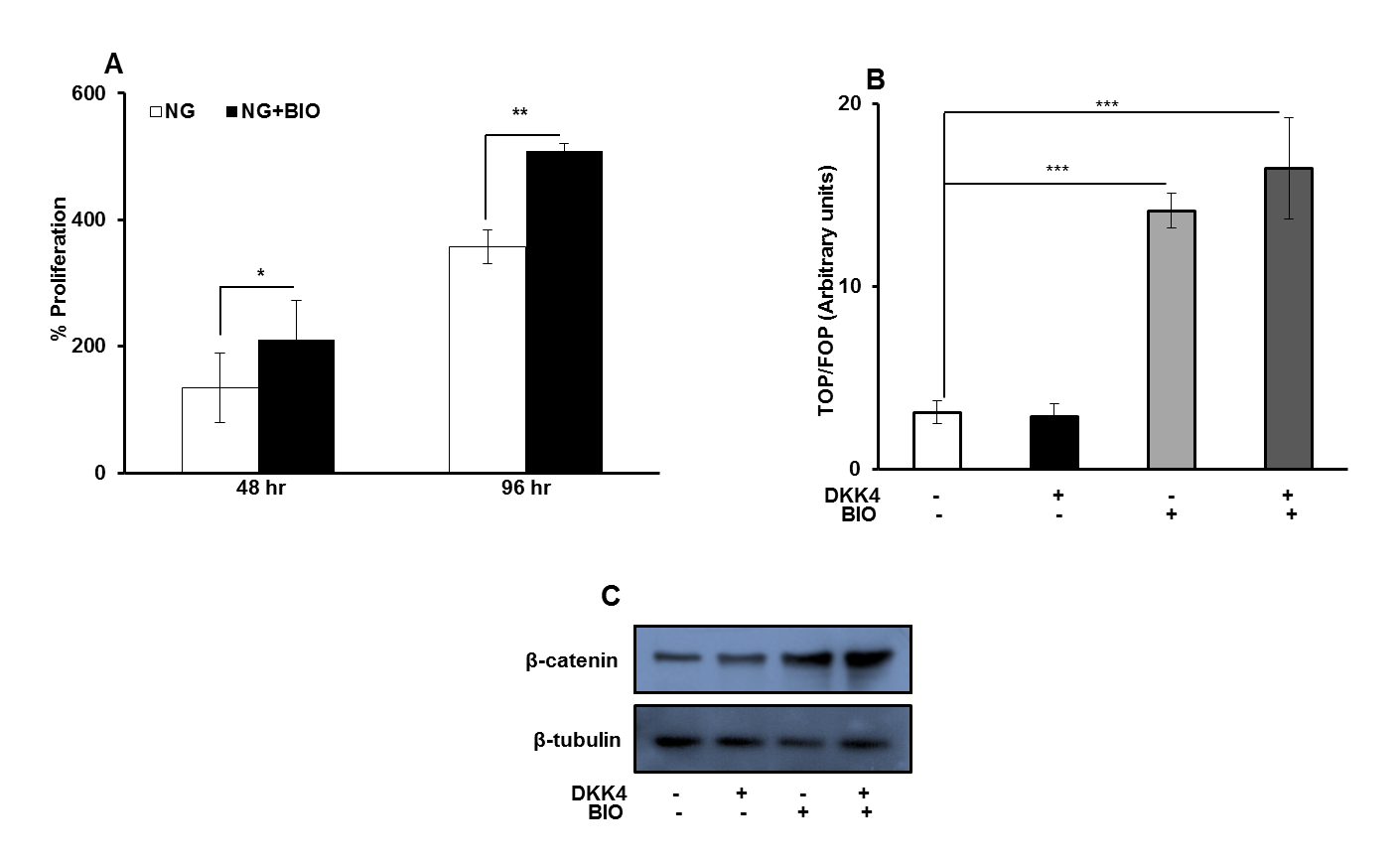
**

**Supplementary Figure 4. BIO treatment increases cellular β–catenin and enhances proliferation of cells in NG.**

(a) HepG2 cells were cultured in NG and NG+BIO for 48 hr and 96 hr and percentage proliferation were determined by MTT assay. Bar graph represents mean±SD of an experiment done in triplicate (*P<0.05, **P<0.001).

(b) TCF reporter activity assay in HepG2 cells cultured in NG, NG+BIO, NG+DKK4 and NG+BIO+DKK4 protein for 16 hr. The luciferase intensities were normalized with Renilla intensities and represented as ratio of TOP/FOP. Bar graph represents mean±SE of three independent experiment (***P<0.0001).

(c) Immunoblotting for β-catenin in HepG2 cells cultured in NG, NG+BIO, NG+DKK4 and NG+BIO+DKK4 protein for 16 hr.


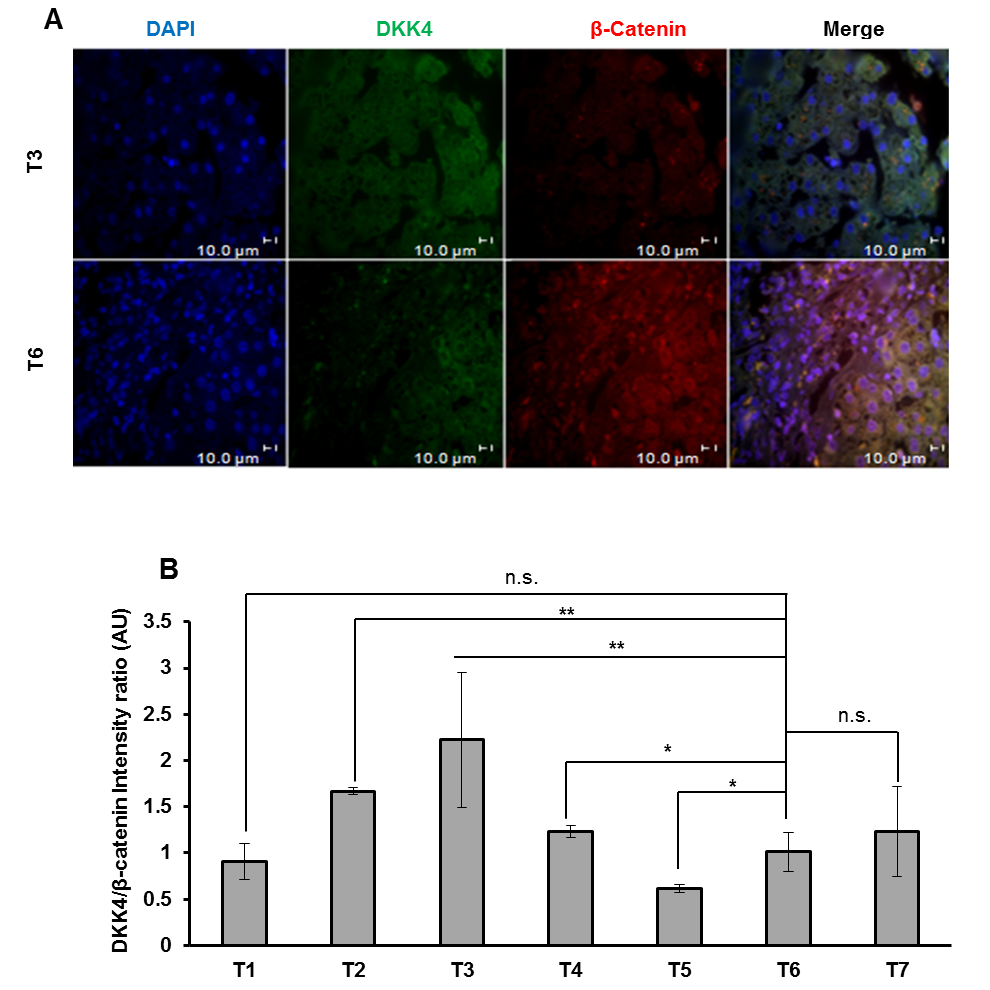


**Supplementary Figure 5. DKK4 and β–catenin protein expression in HCC human samples.**

(a) Representative panel showing immunohistochemical analysis of DKK4 and β–catenin in human HCC tissues with and without diabetes (T6 and T3).

(b) Bar graphs represent mean±SD ratio between fluorescent intensities of DKK4 and β-catenin proteins in three different sections of each human HCC sample (*P<0.05, **P<0.001, ***P<0.0001), n.s. represents non significant P value (P>0.05).


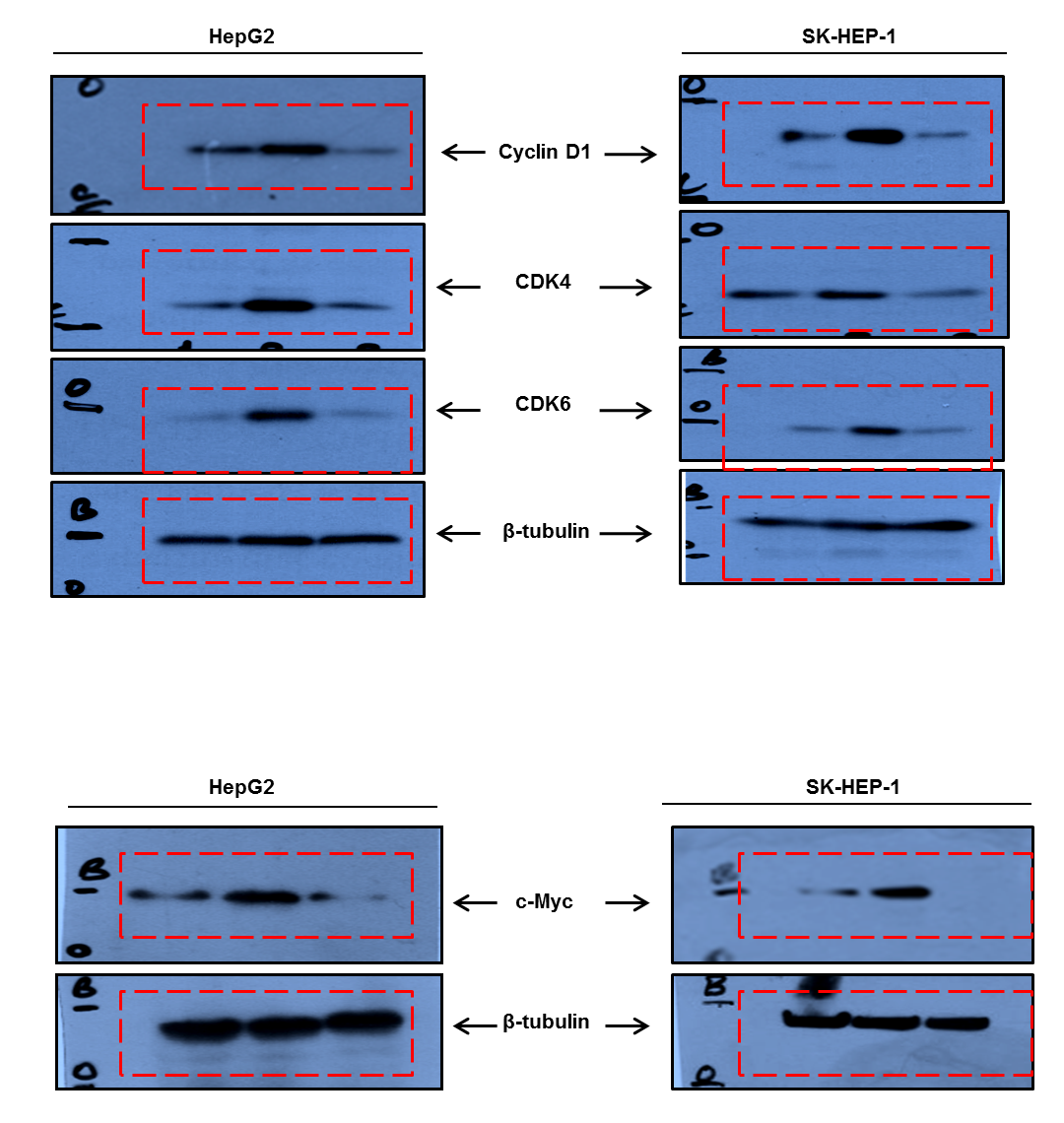


**Supplementary Figure 6. Full length blots of Cyclin D1, CDK4, CDK6, β-tubulin and c-Myc.**


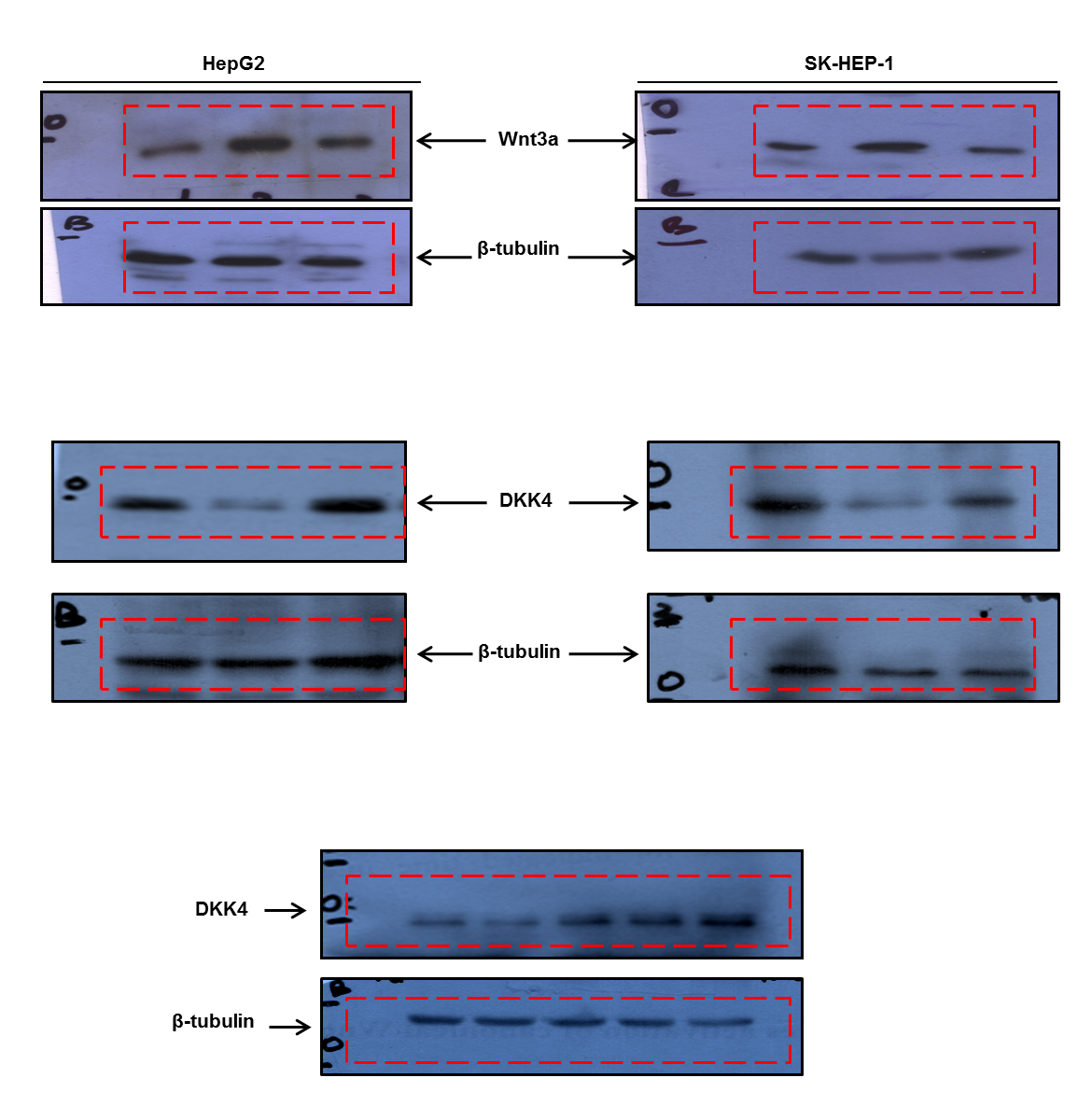


**Supplementary Figure 7. Full length blots of Wnt3a, DKK4 and β-tubulin.**

**
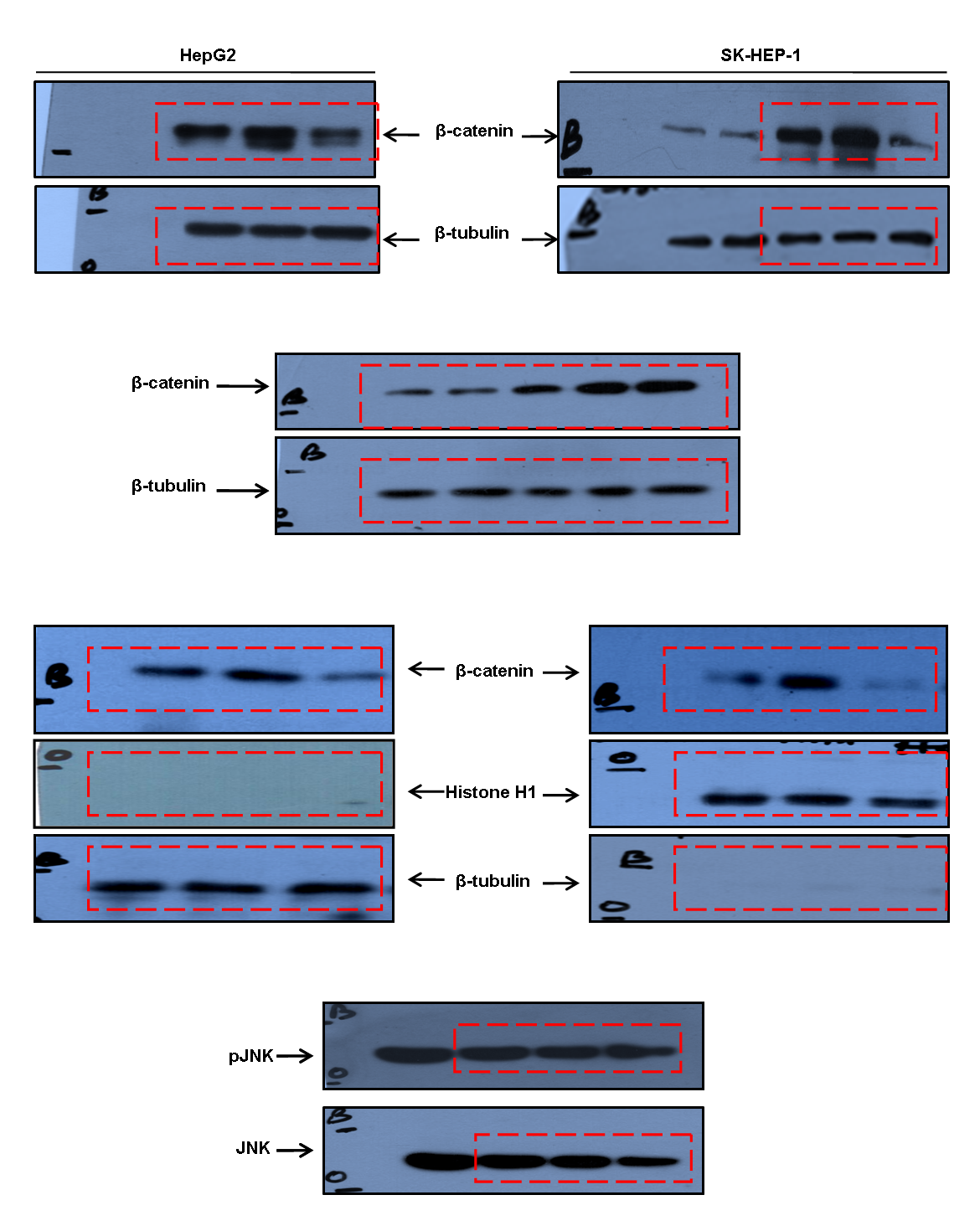
**

**Supplementary Figure 8. Full length blots of β-catenin, Histone H1, pJNK, JNK and β-tubulin.**


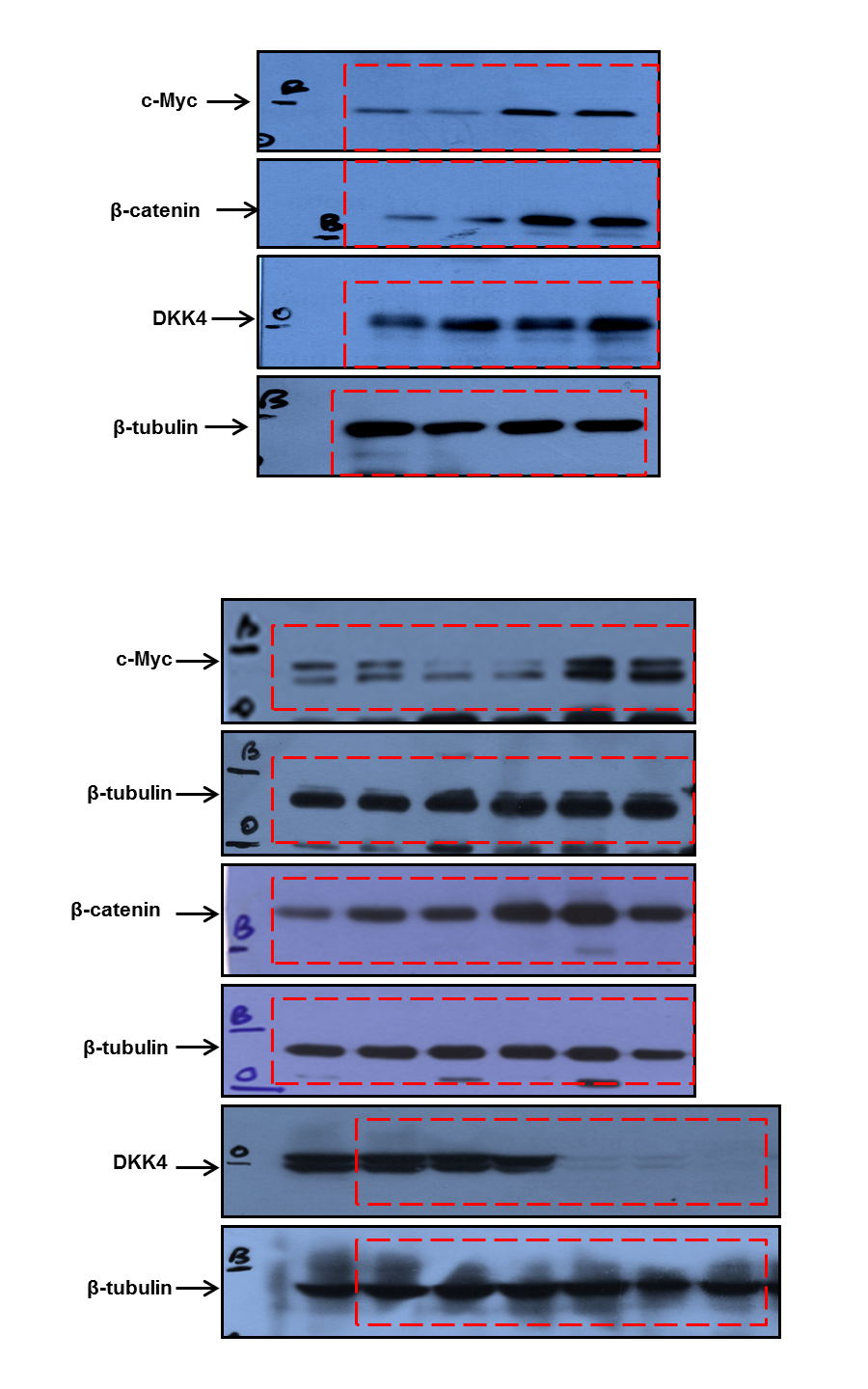


**Supplementary Figure 9. Full length blots of c-Myc, β-catenin, DKK4 and β-tubulin.**


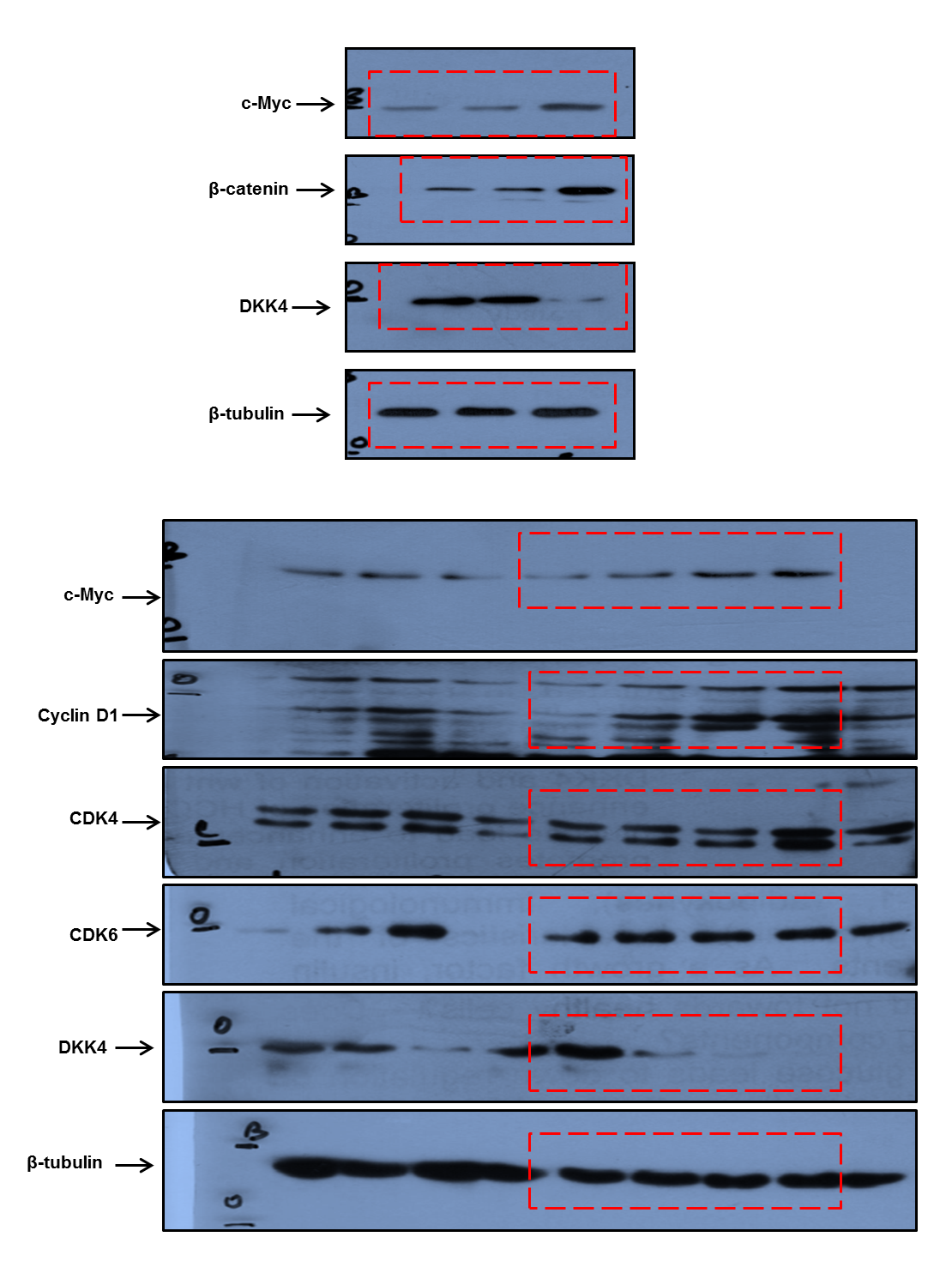


**Supplementary Figure 10. Full length blots of c-Myc, β-catenin, Cyclin D1, CDK4, CDK6, DKK4 and β-tubulin.**

**Supplementary Table.** Physiological parameters observed in human HCC tumors.

| Tumor sample | Tumor 1 | Tumor 2 | Tumor 3 | Tumor 4 | Tumor 5 | Tumor 6 | Tumor 7 |
| --- | --- | --- | --- | --- | --- | --- | --- |
| Age | 33 | 62 | 60 | 68 | 62 | 63 | 73 |
| Gender | Male | Male | Male | Male | Male | Male | Male |
| HBV/C | HBV | HBV | No | HBV | HBV | No | No |
| NASH | No | No | No | No | No | Yes | No |
| Diabetes | No | No | No | No | No | Yes | No |
| Tumor grade | Poor diff | Mod diff | Mod diff | Mod diff | Poor diff | Mod diff | Mod diff |
| Liver cirrhosis | Yes | No | No | No | No | No | Yes |

* Note: Mod=moderately, diff=differentiated.
